# Empathy in Workplace Relationships: Experiences of Generation Z Nurses with Colleagues and Nurse Managers

**DOI:** 10.3390/healthcare14152405

**Published:** 2026-08-05

**Authors:** Aytolan Yıldırım, Ayfer Aydın, Merve Ertunç Soycan, Ritin Fernandez, Tosin Popoola

**Affiliations:** 1 Department of Nursing, Faculty of Health Sciences, Istanbul Atlas University, Istanbul 34408, Türkiye; ayfer.aydin@atlas.edu.tr; 2 School of Nursing, Istanbul University, Istanbul 34116, Türkiye; merve.ertunc@istanbul.edu.tr; 3 School of Nursing and Midwifery, University of Newcastle, Callaghan, NSW 2308, Australia; ritin.fernandez@newcastle.edu.au (R.F.); tosin.popoola@newcastle.edu.au (T.P.)

**Keywords:** empathy, generation Z, nurses, workplace relationships, qualitative research, nursing management

## Abstract

Background: Empathy is essential for supportive nursing work environments, yet little is known about Generation Z (Gen Z) nurses’ experiences of empathy from colleagues and nurse managers. Aim: To examine Gen Z nurses’ experiences of empathy in workplace relationships with colleagues and nurse managers and the individual, generational, and institutional factors shaping these experiences. Methods: A qualitative descriptive study design was employed. Data were collected between October 2025 and March 2026 through semi-structured, in-depth interviews with 20 Gen Z nurses working in diverse clinical settings in Turkey. Participants were recruited using purposive and snowball sampling. Interviews were conducted via Zoom, audio-recorded with consent, and transcribed verbatim. Data were analyzed using Braun and Clarke’s reflexive thematic analysis framework. The Consolidated Criteria for Reporting Qualitative Research (COREQ) checklist guided the study reporting. Results: Four main themes were identified: (1) perceptions of empathy among Gen Z nurses, (2) empathy experiences in workplace relationships, (3) factors influencing empathy experiences, and (4) expectations for building an empathic work environment. Empathy was experienced through feeling understood, non-judgmental listening, and emotional and practical support, particularly in peer relationships. Hierarchical communication, perceived intergenerational differences, workload, and time pressure were key factors influencing workplace empathy experiences. Conclusions: Participants described empathy as a relational and context-dependent experience embedded in everyday workplace interactions. Their accounts suggest that supportive, non-hierarchical relationships may facilitate empathic engagement, whereas rigid communication styles and heavy workloads may act as barriers. These findings represent the experiences and interpretations of the Gen Z nurses interviewed and should not be read as objective comparisons between generations. Organizational strategies that support empathic communication and psychologically safe teamwork may help strengthen team cohesion and nurse retention.

## 1. Introduction

Contemporary healthcare environments are increasingly complex, characterized by demanding workloads, emotional intensity, and multidisciplinary team structures. As a result, technical competence alone is no longer sufficient for effective clinical practice. Clinicians must also possess strong communication and relational skills to deliver safe and effective person-centred care. From a nursing perspective, empathy is a core competency that underpins effective communication, collaboration, professional satisfaction, and the sustainability of high-quality care [1,2,3]. Thus, empathy is vital for navigating the hierarchical structures, time pressure, and high emotional burden that have become hallmarks of modern healthcare settings.

In nursing literature, empathy is conceptualized as a multidimensional construct that involves understanding another person’s emotions and thoughts, communicating that understanding, and linking it to an appropriate professional response through cognitive and affective processes [4]. Numerous studies have demonstrated that empathic communication strengthens patient safety, quality of care, patient satisfaction, and the therapeutic relationship [5,6]. However, empathy is not limited to the nurse–patient relationship and may also shape professional relationships with colleagues and managers [7,8,9]. In this study, empathy refers specifically to attempts to understand another person’s perspective or emotional experience and to communicate or act on that understanding. Psychological safety refers to feeling able to speak, ask questions, or acknowledge difficulties without fear of humiliation or punishment, whereas organizational climate and collegial support describe broader features of the work environment. These constructs may facilitate or result from empathic interaction, but they are not treated as synonyms for empathy.

A supportive, empathetic work environment fosters nurses’ professional commitment, intra-team collaboration, and continuity of care [1,10,11,12]. In contrast, a lack of empathy may lead to negative outcomes, including burnout, job dissatisfaction, interpersonal conflict, and an intention to leave the job [13,14].

These effects may be particularly relevant to newly graduated nurses, many of whom belong to Generation Z (Gen Z) and are in the early stages of their professional careers [15,16]. For this study, Gen Z was operationally defined as people born between 1997 and 2012, consistent with the starting point proposed by the Pew Research Center and the range used in recent nursing research [17,18,19]. Because published cut-offs vary, this range is used as a pragmatic sampling definition rather than as a claim that generational boundaries are fixed or universally agreed upon. The experiences of new graduate nurses during workplace adaptation may have long-term implications for professional identity development and career sustainability [4].

Gen Z is often described in the literature as having grown up amid widespread digital technologies and as valuing rapid feedback, open communication, personal boundaries, and psychological safety [20,21,22]. Some studies report that Gen Z respondents place importance on meaningful work, inclusive relationships, and supportive leadership [1,16,23]. Such descriptions should be understood as context-dependent tendencies or socially constructed cohort narratives rather than inherent characteristics shared by every member of an age group. Age, career stage, historical period, culture, and organizational context may overlap with or account for apparent generational patterns [24].

However, these expectations may lead to adjustment difficulties in nursing environments where traditional hierarchical structures prevail and multiple generations work together [25,26]. One key determinant of Gen Z nurses’ intention to leave their jobs is the working environment, in which empathic relationships are weak or entirely disregarded [27,28]. Studies show that young nurses who feel misunderstood, emotionally unsupported, and unheard by managers and colleagues experience a rapid decline in organizational commitment and an increase in turnover intention [29,30]. In workplaces lacking empathy, Gen Z nurses may experience feelings of worthlessness, burnout, and emotional alienation due to heavy workloads, non-constructive critical feedback, and hierarchical communication styles [31]. Because Gen Z nurses value meaningful relationships, psychological safety, and mutual respect, empathic leadership and supportive team relationships are fundamental to their retention in the profession.

Some studies suggest that persistent lack of support and difficult working conditions may contribute to early turnover among younger or newly qualified nurses [32,33,34]. However, attributing communication patterns, emotional awareness, or empathy to generational membership alone risks obscuring variation within Gen Z and the influence of professional experience, workload, team norms, and organizational culture. Accordingly, the present study uses the generational label to delimit the participant group and explore how participants themselves interpret age- and cohort-related experiences; it does not assume that Gen Z nurses are inherently more empathic, emotionally aware, or communicatively skilled than older nurses.

Most existing empathy research focuses on nurse–patient relationships, whereas empathy in relationships with colleagues and managers has received less attention [10,18,35]. Relevant qualitative evidence nevertheless shows that newly qualified nurses’ workplace experiences are shaped by communication, recognition, team relationships, managerial support, and the transition into professional roles [3,18,26]. Focus-group and interview studies have described the competencies expected of newly graduated Gen Z nurses [2], young nurses’ reasons for leaving organizations [32], new graduates’ transition experiences [3], perceived generational gaps between nurses and managers [18], and Gen X nurse managers’ experiences of leading Gen Z nurses [16]. A qualitative meta-synthesis has likewise shown that new graduate experiences vary across generations and contexts [26], while a recent qualitative study documented the accounts of Gen Z nurses who left hospitals during their first year [34]. Collectively, these studies illuminate workplace support and intergenerational interaction, but they do not specifically explain how Gen Z nurses distinguish and experience empathy in everyday relationships with both colleagues and nurse managers.

Therefore, qualitative inquiry is needed to explore how Gen Z nurses describe empathy in interactions with colleagues and managers and which interpersonal and contextual conditions they perceive as facilitating or constraining it. Such inquiry can preserve the meanings participants assign to their experiences while avoiding assumptions that reported differences constitute stable traits of entire generations.

This study aims to examine how newly graduated Gen Z nurses experience empathy in workplace relationships with their colleagues and managers, and to identify the individual, generational, and institutional factors shaping these experiences. The study is expected to make an original contribution to the nursing literature and to guide practices aimed at developing empathic and supportive work environments.

## 2. Materials and Methods

### 2.1. Study Design and Methods

This study used a qualitative descriptive design to explore Generation Z nurses’ accounts of empathy in workplace relationships with colleagues and nurse managers. Qualitative description was selected because the aim was to provide a comprehensive, practice-oriented account of participants’ experiences in language that remained close to their own descriptions, rather than to identify the essential structure of a phenomenon or develop a hermeneutic interpretation [36,37]. The design was informed by a relational and context-dependent understanding of empathy in nursing, in which empathy is shaped through interpersonal interaction, professional roles, hierarchy, generational positioning, and organizational conditions. This conceptual orientation served as a sensitizing perspective during development of the interview guide but was not imposed as a predetermined coding framework.

### 2.2. Participants

Inclusion Criteria: Participants were required to have at least 3 months of clinical experience, meet the study’s operational definition of Gen Z (born between 1997 and 2012), and volunteer for the study.

Exclusion Criteria: Participants were excluded if they (1) had less than three months of clinical nursing experience, (2) did not belong to Gen Z (i.e., were born before 1997 or after 2012), (3) were not currently working in a clinical setting, (4) were in purely administrative or academic roles without direct patient care responsibilities, or (5) were unwilling or unable to participate in an in-depth interview conducted in Turkish.

### 2.3. Settings

The study was conducted in Turkey and included nurses employed in university hospitals, private/foundation hospitals, and training and research hospitals.

### 2.4. Recruitment

Purposive sampling was initially used to recruit nurses who met the eligibility criteria and could provide information-rich accounts relevant to the study aim. Variation was sought across hospital type, clinical unit, length of experience, and educational background. Snowball sampling was then used to reach eligible nurses beyond the researchers’ immediate contacts and across different institutions. Initial participants were asked to suggest other nurses who met the eligibility criteria and might be interested in participating in the study. The referred nurses were subsequently approached directly by the researchers rather than being contacted or recruited by the initial participants on behalf of the research team. Potential participants were approached individually by A.Y. or A.A. through professional networks, institutional contacts, and participant referrals; eligibility was checked before enrolment, and participation was voluntary. No participant was in a supervisory or dependent relationship with the interviewer. Combining these strategies was appropriate for reaching a specific early-career population across multiple settings; however, recruitment through professional networks and referrals may have introduced selection bias. Nurses with particularly positive or negative workplace experiences, or those more comfortable discussing empathy, may have been more willing to participate. This possibility was considered during analysis and is acknowledged as a limitation.

### 2.5. Data Collection

Data were collected between October 2025 and March 2026 after ethics approval. A.A., an academic nurse researcher experienced in qualitative interviewing, conducted all individual interviews via Zoom. A.A. had no managerial authority over participants and did not work in their clinical units. Before each interview, A.A. explained the study, confirmed eligibility, obtained written and verbal consent, and reminded participants that participation was voluntary. A semi-structured guide was used consistently, while follow-up prompts enabled clarification and elaboration. Interviews were conducted in Turkish, lasted approximately 35–45 min, were audio-recorded with permission, and were transcribed verbatim. Field notes recorded contextual observations and initial analytic reflections after interviews. At the end of each interview, participants were invited to clarify or add anything they considered important. A.A. also summarized the main points discussed and invited participants to confirm, correct, or further clarify the account; no substantive corrections or changes were requested. Transcripts were not returned to participants, and formal member checking of the final themes was not undertaken because the themes were developed as interpretive outputs of reflexive thematic analysis rather than as findings requiring participant validation. Credibility was instead supported through researcher reflexivity, team-based analytic review, an audit trail, and illustrative quotations.

Sample adequacy was evaluated iteratively during data collection and preliminary analysis rather than determined by a numerical rule. After each interview, the researchers reviewed the transcript, field notes, provisional codes, and areas requiring further exploration. The initial interviews generated the most descriptive codes (code saturation), whereas later interviews were assessed for whether they added nuance, variation, or alternative interpretations to developing patterns (meaning sufficiency). By interview 18, the coding framework was stable; interviews 19 and 20 repeated established patterns and enriched examples but did not materially alter the interpretation or thematic structure. Recruitment therefore ended at 20 participants. Because saturation is contested in reflexive thematic analysis, this decision is described as sufficient information power and conceptual depth for the focused aim, specific participant group, quality of interview dialogue, and cross-case thematic analysis, rather than as proof that all possible meanings were exhausted [38,39,40]. The interview guide was developed from the literature and aligned with the study aim. Examples included: “How do you define empathy?” “Can you describe an experience of empathic interaction with your colleagues?” “Can you describe an experience of empathic interaction with your managers?” “In what ways do nurse managers influence your experience of empathy in the workplace?” “What are the main barriers to empathy in your workplace?” and “As a Gen Z nurse, how would you describe your approach to empathy?”

### 2.6. Data Analysis

Reflexive thematic analysis followed Braun and Clarke’s six phases: (1) familiarization, (2) generating initial codes, (3) constructing candidate themes, (4) reviewing themes, (5) defining and naming themes, and (6) producing the report [41,42]. Analysis was conducted on the original Turkish transcripts. Three researchers (A.Y., A.A., and M.E.S.) repeatedly read the transcripts and independently generated initial semantic and, where warranted, interpretive codes relevant to the research question. They then met to compare interpretations, discuss assumptions and divergent readings, and collaboratively refine the coding framework and candidate themes. Coding was iterative: codes were added, merged, renamed, or removed as analysis progressed, and candidate themes were checked against coded extracts and the complete dataset. Differences were treated as opportunities for reflexive discussion rather than resolved through inter-rater reliability statistics. Analytic memos and a decision log documented changes to codes, theme boundaries, and links between interpretations and supporting extracts. No dedicated qualitative data-analysis software was used; coding and theme development were undertaken manually. The final thematic structure was reviewed by the full research team for coherence, distinction, and relevance to the study aim, and representative quotations were selected to illustrate both common and differing accounts. This full-team review brought together the insider linguistic and cultural perspectives of A.Y., A.A., and M.E.S. with the more culturally external perspectives of R.F. and T.P., allowing culturally taken-for-granted assumptions and alternative interpretations to be examined reflexively. Analysis was completed in Turkish. After themes were finalized, quotations selected for publication were translated into English by the researchers and reviewed by bilingual experts. Discrepancies were discussed against the Turkish transcripts to preserve semantic meaning and cultural nuance; only the verified English quotations were used in the report.

### 2.7. Trustworthiness

Trustworthiness was addressed using Lincoln and Guba’s criteria [43] of credibility, dependability, confirmability, and transferability. Credibility was supported through in-depth interviewing, attention to variation and disconfirming accounts, repeated engagement with the transcripts, and analytic discussion among researchers. Dependability was enhanced by using a consistent semi-structured interview guide, having all interviews conducted by the same researcher (A.A.), and documenting decisions related to recruitment, interviewing, coding, and theme development. Confirmability was supported by an audit trail, reflexive memos, team review, and quotations that allow readers to examine the relationship between participants’ accounts and the interpretations presented. Transferability was facilitated by describing the settings, eligibility criteria, sample characteristics, and contextual conditions in sufficient detail for readers to judge relevance to other contexts. The Consolidated Criteria for Reporting Qualitative Research (COREQ) checklist guided transparent reporting [44].

### 2.8. Researcher Reflexivity

The research team comprised nurse academics with experience in nursing management, education, and qualitative research. All participants were Turkish nurses. A.Y., A.A., and M.E.S. were from the Turkish cultural context and were familiar with the language, cultural norms, and organizational context of nursing in Türkiye. Their insider perspectives supported sensitivity to culturally situated meanings and nuances related to empathy, professional hierarchy, seniority, communication, and nurse–manager relationships. R.F. and T.P. were not embedded in the Turkish cultural context and contributed more external perspectives during the full-team analytic review. This combination of insider and outsider perspectives was used as a reflexive resource: the culturally embedded researchers clarified linguistic and contextual nuances, while the culturally external researchers questioned potentially taken-for-granted assumptions and encouraged consideration of alternative interpretations.

These backgrounds supported contextual understanding but could also shape expectations about hierarchy, generational relations, and empathy. The research team also spanned several generational cohorts: A.Y. was a member of the Baby Boomer generation; A.A. and R.F. were members of Generation X; and T.P. and M.E.S. were Millennials. No member of the research team belonged to Generation Z. During analysis, the researchers considered how their own generational positions might influence their interpretations of participants’ expectations regarding feedback, respect, emotional expression, workplace communication, seniority, and managerial support. Differences in cultural and generational positioning were discussed as analytic resources rather than used to establish consensus or validate a single interpretation.

Before and during analysis, researchers recorded assumptions, discussed how professional position and generational identity might influence questioning and interpretation, and actively examined accounts that challenged preliminary patterns. A.Y., A.A., and M.E.S. undertook the initial analysis of the original Turkish transcripts, after which the full research team reviewed the developing thematic structure. This process enabled preliminary interpretations to be examined and challenged across cultural, professional, and generational positions before the themes were finalized. At the time of the study, all members of the research team held academic roles, and none had a concurrent clinical role. Nevertheless, their previous clinical nursing experience and current academic positions were acknowledged as factors that could shape their sensitivity to professional hierarchy, leadership, education, and workplace relationships. A.A. did not work in the participants’ clinical units and had no managerial or supervisory relationship with them. The interviewer used open, non-leading prompts and maintained a reflexive journal. Themes were framed as participants’ situated perceptions rather than objective attributes of Gen Z nurses, experienced nurses, or nurse managers.

### 2.9. Ethical Considerations

Ethical approval was obtained from the Istanbul Atlas University’s Non-invasive Scientific Research Ethics Committee (Date: 2 October 2025, No: 08/70). Interviews were conducted after receiving verbal and written consent. Participants were informed that they had the right to withdraw their consent at any time without any justification.

## 3. Results

### 3.1. Participant Characteristics

The mean age of the participating nurses (*n* = 20) was 25.25 years (range: 23–29); 85% were female, 75% held a bachelor’s degree, 90% were single, and none had children. Participants most often worked in training and research hospitals (40%) and intensive care units (40%). The most common length of work experience was 3 months–1 year (40%). Half of the nurses (50%) had received empathy training; among those trained, 90% reported receiving it during undergraduate education. Demographic characteristics are presented in Table 1.

### 3.2. Themes

**Figure 1:**
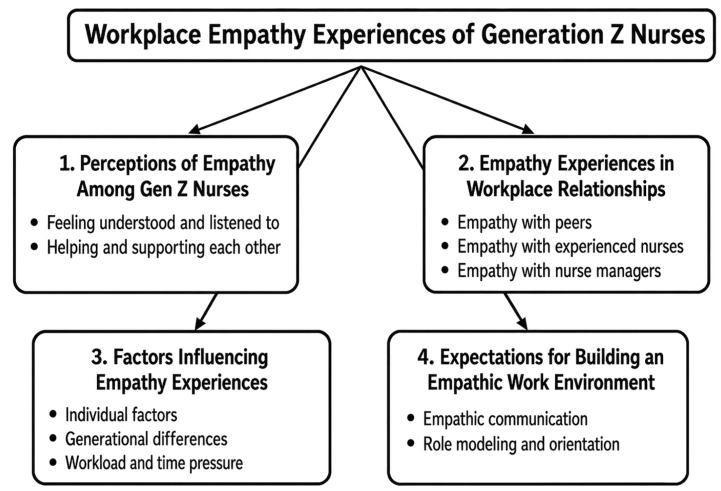
Summary of the final themes and subthemes identified through manual reflexive thematic analysis.

Four main themes were identified: (1) perceptions of empathy among Gen Z nurses, (2) empathy experiences in workplace relationships, (3) factors influencing empathy experiences, and (4) expectations for building an empathic work environment. Figure 1 presents an overview of the four themes and their associated subthemes. Examples of quotes related to the identified themes, subthemes, and participant quotes are presented in Table 2.

#### 3.2.1. Perceptions of Empathy Among Gen Z Nurses

Initially, participants conceptualized empathy primarily within the context of patient care. Nurses defined empathy as “putting oneself in the patient’s shoes” and “trying to understand what the patient might be feeling.” They viewed an empathic approach as a fundamental requirement of nursing. In nurse–nurse and nurse–manager relationships, they expressed these definitions through behavioral and experiential indicators: attempting to understand another person’s perspective, listening without judgment, acknowledging emotions, and responding sensitively. Participants also described helping, feeling supported, trust, and freedom to speak. Analytically, perspective-taking, emotional recognition, and communicated understanding were coded as empathy per se; practical help was interpreted as a possible behavioral expression of empathy. Trust, psychological safety, collegial support, and workload were treated as related outcomes or contextual conditions rather than as empathy itself. This distinction guided the interpretation of the themes that follow.

##### Feeling Understood and Listened to

Almost all nurses associated empathy with active, non-judgmental listening. They emphasized that being listened to without judgment was especially important when sharing the difficulties they experienced in the clinical setting, and they defined an empathetic approach as “*feeling truly understood*.” They reported that such experiences helped reduce their emotional burden and made work-related stress more manageable. For the participants, empathetic listening was not necessarily perceived as a problem-solving process; rather, the acceptance of the individual’s experiences as valid and understandable constituted the core of the empathetic experience.

*“When I tell my colleagues that I can’t keep up with my tasks, they listen to me without judging. I think that’s empathy—they understand me better, and I understand them better, because we have gone through similar processes and experienced the same things.”*

(N2, 1 year 4 months’ experience)

*“I don’t always expect a solution. Sometimes I just want to feel that what I’m going through is normal and understandable. Even that alone is enough to reduce work-related stress.”*

(N10, 1 year 6 months’ experience)

##### Helping and Supporting Each Other

Participants reported feeling empathy when colleagues offered help without being asked, particularly after completing their own tasks and assisting with complex patient care. They interpreted this as an indication that their colleagues were aware of the difficulty of their situation and genuinely understood it. They also emphasized that they reciprocated this behavior by supporting their colleagues in similar situations, thereby demonstrating empathy through action.

*“For me, a colleague who notices I am overwhelmed and comes to help without waiting to be asked shows true empathy.”*

(N1, 3 years 9 months’ experience)

*“For me, empathy is not only about words, but also about actions. When someone comes to help me exactly when I need it, it shows that they truly understand my situation.”*

(N3, 6 months’ experience)

#### 3.2.2. Empathy Experiences in Workplace Relationships

This theme illustrates how participants experienced empathy in their relationships with colleagues and managers in the workplace and how these experiences varied across relational levels. These findings highlight that empathy is a dynamic process shaped not only by individual skills but also by relational context, power dynamics, and the degree of reciprocity within interactions. Participants’ situated interpretations of specific workplace encounters should be understood as the meaning behind these accounts, rather than as independently verified assessments of the empathic capacities, intentions, or communication styles of experienced nurses or nurse managers.

##### Empathy with Peers

The majority of participants reported experiencing the strongest sense of empathy in interactions with colleagues from their own age group. Compared to senior or managerial relationships, peer interactions were described as more naturally empathic, shaped by shared professional challenges and emotional experiences that enabled immediate and intuitive understanding.

Rather than being expressed primarily through explicit actions, peer empathy was characterized by an implicit sense of being understood without verbalization. Participants noted that colleagues could quickly recognize their emotional states and needs, fostering a spontaneous, embedded form of empathy in everyday interactions.

Additionally, peer relationships were perceived as more egalitarian and less subject to hierarchical pressures, facilitating open communication and reducing fear of judgment. This environment allowed participants to express vulnerability more comfortably, making empathy more accessible, mutual, and sustainable.

Empathic relationships among peers were also perceived as reducing feelings of loneliness, alleviating the emotional burden of work, and strengthening team solidarity. In this sense, empathy functions not only as emotional sharing but also as a protective factor that supports psychological resilience in demanding clinical environments. In particular, peer-based empathy is characterized by reciprocity and the absence of hierarchical barriers, enabling more open, balanced, and supportive interactions.

*“I feel my generation is more empathetic—we try to help and understand patients more. More experienced nurses sometimes seem less patient and understanding, especially in the wards.”*

(N3, 6 months’ experience)

*“Senior nurses sometimes focus more on criticism, while my peers try to find solutions. When I make a mistake, they focus on fixing it rather than blaming me, which makes me feel supported.”*

(N8, 2 years’ experience)

##### Empathy with Experienced Nurses

Gen Z nurses reported that they often perceive empathy in their interactions with experienced colleagues as limited, distant, and at times challenging. Participants indicated that encountering statements such as “*we worked under much more difficult conditions” or “you should have learned this by now*” not only hindered communication but also created emotionally distressing experiences. Such expressions reinforce feelings of being misunderstood, undervalued, and having their efforts overlooked. Participants further emphasized that experienced nurses tend to rely heavily on past experience, interpret situations within a more rigid, serious framework, and are therefore less open to new approaches. This was experienced as their perspectives being insufficiently acknowledged and, at times, underestimated or dismissed. In addition, the task-oriented and work-focused approach of senior nurses was reported to limit empathic interaction by pushing the emotional dimension into the background.

Moreover, the tendency of experienced nurses to adopt a fault-finding rather than a supportive approach was perceived as a lack of empathy and described as a form of communication in which emotional needs were overlooked. In this context, empathy for Gen Z nurses was associated more with being judged than understood, and more with being criticized than supported. Interactions lacking empathy were reported not only to weaken interpersonal relationships but also to further deepen the generational gap.

*“Sometimes, when I say that I am struggling with something or ask for help, I receive responses like, ‘We worked under much more difficult conditions.’ Rather than motivating me, these kinds of approaches make me feel undervalued. It feels as if the difficulties I experience are being minimized and that I am not truly understood.”*

(N8, 2 years’ experience)

*“When I make a mistake or haven’t yet learned something, being told, ‘you should have learned this by now,’ really wears me down. At that moment, when I expect support, being criticized makes me feel inadequate and makes communication even more difficult.”*

(N2, 1 year 4 months’ experience)

##### Empathy with Nurse Managers

Gen Z nurses reported that they primarily communicate with unit-level nurse managers, while their interactions with hospital administrators remain limited. Their experiences of empathy in relationships with nurse managers were perceived as two-sided and variable.

Managers who were perceived as empathetic were described as understanding, approachable, and solution-oriented leaders who listen to employees’ concerns and actively seek solutions. In interactions with such managers, nurses reported feeling safer, more valued, and supported. In this context, empathy was not limited to understanding emotions but also involved collaboratively seeking solutions and taking employees’ needs into account.

In contrast, distant and non–solution-oriented managerial attitudes were reported to significantly weaken empathy. Participants stated that some managers did not communicate sufficiently with staff, made little effort to understand problems, and often adopted a purely task-oriented approach.

Some participants described these environments, where empathy was lacking, as emotionally exhausting and, at times, as a form of mobbing. The absence of empathetic support not only made daily work experiences more difficult but also weakened nurses’ sense of belonging to their unit and institution. Accordingly, some nurses reported considering or deciding to change their unit or workplace in response to these negative experiences.

Overall, for Gen Z nurses, empathy in relationships with nurse managers emerged as either a supportive and empowering factor or, depending on the manager’s approach, as an element that creates distance and weakens communication. Participants also emphasized that the number of managers demonstrating an empathetic approach remains limited, indicating a significant need for improvement in this area.

*“We mostly communicate with our unit supervisors. We don’t really have much interaction with hospital managers. But if our unit manager is understanding, it really makes our job easier.”*

(N7, 10 months’ experience)

*“I don’t want to work long-term in an environment where I feel misunderstood. That’s why, like some of my colleagues, I considered changing my unit. There was clearly mobbing in the institution before. The work I did was constantly criticized. There was no empathetic environment at all. Because it’s not just the job itself—the work environment is also very important.”*

(N16, 2 years 6 months’ experience)

#### 3.2.3. Factors Influencing Empathy Experiences

Participants stated that empathy was influenced not only by individual characteristics but also by organizational and structural conditions. Empathy was shaped by both personal capacity and the conditions provided by the work environment.

##### Individual Factors

Participants emphasized that an empathic approach is not solely a skill developed through professional experience; rather, it is shaped by innate tendencies, family values, and individual personality traits. Empathy was described as emerging more naturally and intuitively in some individuals, whereas it remained more limited in others.

In this context, nurses reported that characteristics such as individual sensitivity, emotional awareness, patience, understanding, and the ability to recognize others’ emotions directly influence empathic capacity. Additionally, upbringing, family communication patterns, and past life experiences were identified as key factors in the development of empathic tendencies. Despite working in the same clinical environments under similar conditions, participants attributed variations in empathy levels to individual differences.

Overall, this subtheme positions empathy not merely as a learned professional skill but as a multidimensional construct shaped by an individual’s personal history and internal characteristics.

*“I do not see empathy as merely a professional skill. Although we receive the same education, not everyone demonstrates the same level of empathy. This suggests that personality traits play an important role. Qualities such as being patient, understanding, and genuinely willing to listen vary from person to person.”*

(N9, 6 months’ experience)

*“I think empathy is shaped by family upbringing, social experiences, and coping with challenges, which can help individuals develop a stronger sense of empathy.”*

(N1, 3 years 9 months’ experience)

##### Generational Differences

Participants frequently interpreted their empathy experiences through a generational lens. Many perceived relationships with colleagues of a similar age or career stage as more empathic, explaining this in terms of shared experiences and a sense of being understood. These accounts describe participants’ interpretations of particular interactions; they do not establish that same-generation colleagues are objectively or consistently more empathic.

By contrast, some participants perceived empathy from more experienced or older nurses as more limited. They described particular interactions as rigid, rule-oriented, or framed through comparisons with earlier working conditions. Some also reported feeling labeled as “relaxed,” “irresponsible,” or “not sufficiently work-focused.” These descriptions represent how the interviewed Gen Z nurses made sense of these encounters and cannot determine the intentions, perspectives, or empathy of experienced nurses who were not interviewed.

Participants further attributed some tensions to perceived differences in lifestyle, work–life balance, and professional values. They sometimes contrasted what they viewed as experienced nurses’ work-centered orientation with their own preference for balance and flexibility. Because career stage, organizational role, workplace history, and current conditions were not independently compared, these accounts are presented as perceived intergenerational differences rather than verified cohort effects.

Overall, this subtheme shows how participants used generational categories to interpret differences in values, experiences, and expectations. The findings indicate that these interpretations could facilitate solidarity with peers or contribute to distance from older colleagues; they should not be generalized as fixed characteristics of Gen Z or experienced nurses.

*“Sometimes they see us as irresponsible or too relaxed, but that’s not actually the case. We simply value having a life outside of work as well. Since they are more work-focused, they cannot fully understand this, and it creates a distance between us.”*

(N4, 1 year’s experience)

*“It has an impact, especially in the workplace where generational differences are clear. Older nurses say they handled heavier workloads without complaint and expect the same today, but I don’t think that’s fair. Each generation has different conditions, so the past shouldn’t be judged the same way now.”*

(N18, 3 months’ experience)

##### Workload and Time Pressure

The subtheme “Workload and time pressure” highlights heavy workload and persistent time constraints as key structural factors that limit empathy. Participants reported that in fast-paced, efficiency-driven work environments, empathic interactions are often deprioritized. The ongoing pressure to complete tasks alongside increasing responsibilities makes it difficult for nurses to both recognize their own emotions and respond sensitively to patients’ emotional needs. Gen Z nurses emphasized that the expectation to perform multiple tasks simultaneously, flawlessly and without error, creates significant stress and burnout when combined with time pressure. This situation rapidly depletes empathic capacity, particularly during fatigue, and results in emotional withdrawal. In addition, the constant expectation of high performance and the perception of being criticized were reported to reinforce feelings of mobbing among some participants, contributing to increased interpersonal distance. Within this context, empathy is experienced not only as an individual skill or willingness but also as a process shaped by the time, energy, and psychological space provided by working conditions. Heavy workload and time pressure emerge as significant structural barriers that weaken the emotional dimension of care and limit empathic interactions.

*“Sometimes we are so busy that there is really no time to understand how someone feels. We just focus on getting the work done.”*

(N2, 1 years’ experience)

*“There is always a rush to keep up. So, no one asks, ‘Are you okay? There is simply no time to show empathy.”*

(N7, 8 months’ experience)

#### 3.2.4. Expectations for Building an Empathic Work Environment

This theme reflects participants’ expectations and recommendations for a more empathic work environment. They described empathy as something that should be supported at the organizational level through communication practices, managerial behavior, and orientation. In interpreting these accounts, empathic understanding and response were distinguished from broader organizational conditions: psychological safety, supportive leadership, collegial support, and institutional policies were understood as contexts that may enable empathy rather than as empathy itself.

##### Empathic Communication

Gen Z nurses reported that they expect a more understanding, non-judgmental, and mutually respectful communication style in the workplace. Participants emphasized that empathic communication is not limited to the transfer of information; rather, it involves an interactive process that involves active listening, understanding, and acknowledging individuals’ emotions and experiences.

Gen Z nurses also stated that labeling and generalizing attitudes toward them, such as being described as “too relaxed”, undermine empathic communication. Instead, they highlighted the need for a more open and supportive communication style that recognizes individual differences. They expressed that a more understanding approach enhances both professional motivation and team relationships. In this context, empathic communication not only improves interpersonal relationships but also serves as a fundamental expectation that fosters intergenerational understanding, reduces prejudice, and contributes to a safer work environment.

*“We are not lazy or too relaxed; we just expect our differences to be accepted. We simply want to be understood. When they listen to us, everything becomes easier.”*

(N4, 1 year’s experience)

*“Empathy is not just about talking; it is about truly trying to understand the other person. When I feel that, I perform better.”*

(N2, 1 years’ experience)

##### Role Modeling and Orientation

Gen Z nurses emphasized that working with a preceptor nurse, having an effective orientation process, and receiving regular in-service training contribute not only to the development of clinical knowledge and skills but also to learning how to demonstrate an empathic approach.

In this process, the attitudes and behaviors of managers and experienced nurses who are perceived as role models play a decisive role. Working with experienced nurses who communicate empathetically and adopt a supportive, understanding approach was reported to shape participants’ communication styles and facilitate the internalization of empathy. Conversely, non-empathetic or judgmental attitudes may negatively affect the learning process.

In this context, role modeling and orientation processes are not merely technical stages that ensure professional adaptation; they are also fundamental processes through which empathic values are transmitted, professional identity is shaped, and a supportive team culture is established. For Gen Z nurses, structuring these processes in a strong, intentional manner is considered critical to sustaining an empathy-based work environment.

*“I remember when I first started, my preceptor nurse was really supportive; she didn’t just teach me the tasks, she also showed me how to approach people, and that was very valuable for me.”*

(N7, 10 months’ experience)

*“The attitude of the nurse manager is very important. If they are understanding and supportive, the same approach spreads throughout the team.”*

(N15, 5 years’ experience)

## 4. Discussion

This study provides an in-depth account of how the Gen Z nurses in this study perceived and experienced empathy in workplace relationships. Participants described empathy as a relational and context-dependent process involving perspective-taking, non-judgmental listening, emotional recognition, and communicating understanding. These findings extend the literature by illustrating the everyday behaviors through which this participant group recognized empathy. Importantly, empathy should not be treated as interchangeable with every positive workplace interaction. Psychological safety, teamwork, collegial support, organizational climate, and effective leadership are broader and conceptually distinct constructs. They may create conditions that facilitate empathic communication, may emerge as consequences of empathic interaction, or may coexist with empathy without constituting empathy itself. Consistent with qualitative studies of newly graduated nurses, participants valued emotional recognition, open communication, and feeling safe to express concerns [34,45]. Their emphasis on listening and validation is also compatible with relational models that conceptualize empathy as a dynamic interactional process [46,47].

Participants frequently interpreted practical assistance as evidence that a colleague had noticed, understood, and responded to their difficulty. In this analysis, practical help was therefore regarded as a possible behavioral expression of empathy only when it appeared to be grounded in recognition of another person’s emotional or situational experience. By contrast, routine task-sharing, teamwork, and general workplace support were treated as related but distinct forms of collegial behavior. This distinction is important because a supportive action may occur without explicit perspective-taking, while empathic understanding may also be communicated without practical assistance. Participants most often reported empathy in peer interactions and attributed this to shared experiences, similar workloads, reciprocal relationships, and fewer hierarchical barriers. Thus, the accounts suggest that empathy was embedded within, but not synonymous with, broader experiences of support and psychological safety.

Participants described some interactions with experienced nurses as emotionally invalidating, particularly when criticism or comparisons with past hardships were perceived to minimize their difficulties. However, these accounts should not be interpreted as objective evidence that one generation is more or less empathic than another. Only Gen Z nurses were interviewed, and the study did not include the perspectives of experienced nurses or nurse managers regarding the same interactions. Experienced staff may understand their communication as promoting standards, patient safety, professional responsibility, or resilience, even when newly qualified nurses experience the same communication as unsupportive. The findings therefore represent one group’s situated interpretations and cannot determine the intentions, empathic capacity, or perspectives of the colleagues and managers being described. Qualitative research has nevertheless identified perceived differences in communication, workplace expectations, and relational priorities across age groups and roles [16,18,26]. The present findings illuminate participants’ meaning-making around generational categories rather than stable characteristics of Gen Z or older nurses [24].

Several experiences framed by participants as generational differences may also reflect professional experience, organizational tenure, or career stage. Most participants were in the early years of practice, and newly graduated nurses commonly require guidance, reassurance, constructive feedback, role clarification, and psychological safety while developing clinical confidence. Consequently, the greater sense of understanding reported in peer relationships may partly arise from being at a similar stage of professional transition rather than from generational membership alone. Similarly, the more task-focused communication attributed to experienced nurses may reflect differences in responsibility, workload, accumulated clinical experience, or expectations of independent practice. Because birth cohort, age, career stage, professional experience, organizational role, and historical context were not independently compared, the study cannot establish which of these factors best explains the reported differences.

Perceived generational differences were nevertheless a prominent interpretive frame in participants’ accounts. Some participants associated their own cohort with emotional openness, mutual understanding, and work–life balance, while describing older colleagues as more task- or experience-oriented. These descriptions should be understood as socially and organizationally situated perceptions rather than verified cohort effects. Generational categories have variable boundaries, may encourage stereotyping, and can obscure substantial diversity within age groups; apparent differences may instead reflect age, career stage, period, culture, work setting, or power relations [24]. The findings therefore do not indicate that older nurses lack empathy or that Gen Z nurses are inherently more empathic, emotionally aware, or communicatively skilled. Rather, they suggest that different expectations regarding feedback, support, professional autonomy, and the expression of concern may create relational misalignment in particular workplace encounters. Workload and time pressure were also described as structural constraints on opportunities for empathic engagement, consistent with prior research on emotional capacity under sustained work demands [31,48,49].

Empathy experiences with nurse managers were similarly variable and dependent on participants’ interpretations of leadership behavior. Approachable managers who listened, acknowledged concerns, and responded sensitively were perceived as empathic, whereas distant or predominantly task-focused communication was interpreted as limiting empathy. Solution-oriented leadership, responsiveness, psychological safety, and staff support should not, however, be treated as equivalent to empathy. They are broader leadership and organizational processes that may enable empathic understanding to be expressed and acted upon. These patterns are compatible with literature linking relational or resonant leadership with job satisfaction, empowerment, and retention [1,2,29,33], but the present qualitative data do not establish causal effects. Participants also expected orientation, mentorship, role modeling, and communication practices to support empathic interaction. Such processes may provide the relational space in which perspective-taking, emotional recognition, and responsive communication become possible in daily work [2,9,26].

## 5. Strengths and Limitations

This study has several strengths that enhance its contribution to the nursing literature. First, it provides an in-depth qualitative exploration of empathy from the perspective of Gen Z nurses, a population that remains underrepresented in existing research. By focusing on workplace relationships with both colleagues and nurse managers, the study extends the understanding of empathy beyond the traditional nurse–patient context and highlights its relational and organizational dimensions.

Second, the use of purposive and snowball sampling enabled the inclusion of participants with diverse experiences across different hospital settings, enhancing the richness and variability of the data. The application of a rigorous thematic analysis approach, supported by peer debriefing and adherence to COREQ guidelines, strengthens the credibility and trustworthiness of the findings.

Despite these strengths, several limitations should be considered. First, the study involved a relatively small sample within a single national context, which may limit transferability. Furthermore, the use of purposive and snowball sampling methods, including professional networks, may have introduced selection bias, as nurses with particularly positive or negative workplace experiences, or a greater interest in empathy, may have been more inclined to participate. Consequently, the sample may not accurately represent the full range of experiences among Gen Z nurses. In addition, generational labels are contested social and analytical categories with variable birth-year boundaries. The design cannot disentangle generation from age, career stage, professional experience, historical period, or organizational context [24]; therefore, reported “generational differences” should be read as participants’ interpretations rather than cohort effects.

Second, the reliance on self-reported data collected through semi-structured interviews may introduce social desirability bias. Participants may have emphasized socially acceptable responses or underreported negative experiences. In addition, the use of online interviews may have limited the ability to observe nonverbal cues, which are important components of empathic communication.

Third, although careful translation procedures were implemented, translating interview data from Turkish into English may have resulted in subtle losses of linguistic nuance and cultural meaning, particularly regarding emotional expressions and context-specific interpretations of empathy.

Finally, only Gen Z nurses were interviewed. This is a central interpretive limitation because empathy in workplace relationships is reciprocal and multi-directional. The absence of experienced nurses’ and nurse managers’ perspectives prevents comparison of how the same interactions might be understood by different stakeholders and precludes objective conclusions about intergenerational differences. Participants’ descriptions of older colleagues may not reflect those colleagues’ intentions, constraints, or interpretations. In addition, although reflexive journaling, analytic memos, team discussion, and attention to disconfirming accounts were used, the research team’s nursing, management, educational, and generational assumptions may still have shaped questioning and interpretation. The findings should therefore be read as a reflexively produced account of the participating Gen Z nurses’ situated perceptions rather than a neutral description of other generations. Future multi-perspective studies should include newly qualified nurses, experienced nurses, and nurse managers, and should examine how empathy is distinguished from psychological safety, collegial support, teamwork, and leadership across shared workplace events.

## 6. Implications for Policy and Practice

Given the qualitative design and the perspectives of 20 Gen Z nurses, the findings offer practice-oriented considerations rather than prescriptive policy recommendations. They may inform the development and prospective evaluation of initiatives intended to support empathic workplace relationships.

First, healthcare organizations may consider exploring how empathy-related competencies—such as attentive listening, emotional validation, perspective-taking, and non-judgmental communication—can be incorporated into and evaluated within leadership development. The present findings suggest that managers’ communication and responsiveness may influence how early-career nurses experience workplace relationships; however, the effectiveness of specific training or performance-evaluation strategies should be tested in larger, multi-stakeholder studies.

Second, the findings may inform the development and evaluation of orientation and transition-to-practice programs that include relational communication, constructive feedback, and supportive role modeling. Participants’ accounts indicate that observed behaviors during orientation shaped their perceptions of empathy, but prospective research is needed to determine whether trained preceptorship improves adaptation, well-being, or retention.

Third, perceived intergenerational tensions should be addressed cautiously at the organizational level. The present findings do not verify fixed differences between generations; rather, they suggest that differences in career stage, experience, communication expectations, and professional roles may contribute to misunderstanding. Educational activities that promote perspective-taking, constructive feedback, mutual respect, and dialogue across career stages may help teams examine these differences without reinforcing generational stereotypes.

Fourth, participants’ descriptions suggest that workload and time pressure may restrict opportunities for empathic interaction. Organizations may therefore consider examining workload distribution and the relational demands of clinical work alongside patient-safety and workforce indicators. Decisions concerning staffing models or nurse-to-patient ratios require broader evidence, resource assessment, stakeholder consultation, and prospective evaluation beyond the present study.

Finally, empathy-related behaviors may be considered as a possible focus for future quality-improvement work, provided that empathy remains conceptually distinct from psychological safety, teamwork, and general support. The feasibility and validity of empathy indicators, non-punitive error-management approaches, or recognition programs should be examined with multiple stakeholder groups before routine implementation. This study does not establish that such strategies will improve retention, burnout, or quality of care.

Overall, the findings identify leadership communication, orientation, interprofessional dialogue, workload, and organizational context as areas that may warrant further investigation. Multi-site and multi-perspective studies are required before translating these observations into system-level policy or quality standards.

## 7. Conclusions

From the perspective of the Gen Z nurses interviewed, empathy was described as a relational, behavioral, and context-dependent experience embedded in everyday workplace interactions. Participants primarily recognized empathy through being listened to without judgment, feeling understood, and perceiving a response to their emotional or practical needs.

Participants reported empathy most often in peer relationships, which they attributed to shared experiences and reciprocal interaction. They perceived empathy in relationships with some experienced nurses and nurse managers as more variable and linked these experiences to communication and hierarchy. These findings reflect one participant group’s accounts and do not demonstrate that Gen Z nurses are more empathic, emotionally aware, or communicatively skilled than older nurses. Nor do they establish that experienced nurses or managers are less empathic. Rather, they show how participants interpreted particular workplace encounters through generational, relational, and organizational lenses. Workload and limited time were also described as constraints on opportunities for empathic interaction.

Participants further viewed supportive leadership, orientation, and psychologically safe communication as conditions that may facilitate empathy. These broader organizational constructs are related to, but conceptually distinct from, empathy itself.

Overall, the findings suggest that healthcare organizations may support empathic workplace relationships by encouraging attentive listening, respectful responses, inclusive communication, and adequate relational space within clinical work. Further research incorporating experienced nurses and nurse managers is needed before drawing conclusions about intergenerational differences or their implications for retention and team cohesion.

## Figures and Tables

**Table 1:** General demographic data of nurses (*n* = 20).

Characteristic	*n* (%)
Age (years) Mean Range	25.25 years 23–29 years
Gender Female Male	17 (85%) 3 (15%)
Educational status Bachelor’s degree Postgraduate	15 (75%) 5 (25%)
Marital Status Single Married	18 (90%) 2 (10%)
Child ownership No	20 (100%)
Type of hospital University hospital Private hospital State hospital Training and research hospital	5 (25%) 3 (15%) 4 (20%) 8 (40%)
Specialty area Intensive care units Surgical units Internal medicine units Operating room Cardiology units Pediatric units	8 (40%) 4 (20%) 2 (10%) 2 (10%) 3 (15%) 1 (5%)
Work experience 3 months–1 year 1–3 years 3–5 years	8 (40%) 6 (30%) 6 (30%)
Empathy training Yes No	10 (50%) 10 (50%)
Source of training (*n* = 10) Undergraduate education Course	9 (90%) 1 (10%)

**Table 2:** Experiences of Gen Z Nurses with Colleagues and Nurse Managers.

Themes	Subthemes	Quotes
Perceptions of Empathy Among Gen Z Nurses	Feeling understood and listened to	*“My peers understand me better, and I have less difficulty communicating with them.”* (N1, 3 years 9 months’ experience). *“When a manager said, ‘I understand how pressured you feel right now,’ that really stayed with me.”* (N8, 2 years’ experience).
	Helping and Supporting Each Other	*“If they see I am overloaded and come to help without being asked, that shows empathy.”* (N1, 3 years 9 months’ experience). *“When their cases were over, they came to help me and opened my materials because they knew I was tired.”* (N3, 6 months’ experience).
Empathy Experiences in Workplace Relationships	Empathy with peers	*“We understand each other better because we are all at the beginning of the same journey.”* (N12, 1 years’ experience). “Gen Z nurses understood me better, and I also understood them better.” (N2, 1 year 4 months’ experience).
	Empathy with experienced nurses	*“With more senior nurses, communication felt more serious and rigid.”* (N14, 1 year 6 months’ experience). *“Some experienced nurses say, ‘Don’t you know this yet?’* (N1, 3 months’ experience).
	Empathy with nurse managers	*“Our manager tried to solve my problems and gave me enough time during orientation.”* (N3, 6 months’ experience). *“One manager saw how much we were doing and said, ‘Let me help you so you do not carry more than you should.’”* (N2, 1 year 4 months’ experience).
Factors Influencing Empathy Experiences	Individual characteristics	*“I think empathy is also related to family upbringing; it starts in childhood.”* (N8, 2 years’ experience). *“Character and the people you spend time with shape how empathic you become.”* (N10, 1 year 6 months’ experience).
	Generational differences	*“I think the generational gap makes communication harder.”* (N1, 3 years 9 months’ experience). *“Newly appointed nurses seemed more empathic than older staff to me.”* (N10, 1 year 6 months’ experience).
	Workload and time pressure	*“You can see empathy most clearly in the busiest moments, when the workload is highest.”* (N16, 2 years 6 months’ experience). *“Managers may avoid solutions because they already have their own workload and pressure.”* (N8, 2 years’ experience).
Expectations for Building an Empathic Work Environment	Empathic Communication	*“She was completely solution-focused when we talked about the problem.”* (N7, 10 months’ experience). *“There should be regular, unit-specific communication training for all staff.”* (N1, 3 years 9 months’ experience).
	Role Modeling and Orientation	*“During orientation, she personally came and helped me; that increased my confidence.” (N3, 6 months’ experience).* *“Newcomers often copy what they first see in the unit and treat it as the right way.”* (N1, 3 years 9 months’ experience).

## Data Availability

The data that support the findings of this study are available on request from the corresponding author. The data are not publicly available due to privacy or ethical restrictions.
